# Impact of Depleting Therapeutic Monoclonal Antibodies on the Host Adaptive Immunity: A Bonus or a Malus?

**DOI:** 10.3389/fimmu.2017.00950

**Published:** 2017-08-14

**Authors:** Claire Deligne, Benoît Milcent, Nathalie Josseaume, Jean-Luc Teillaud, Sophie Sibéril

**Affiliations:** ^1^Cordeliers Research Center, INSERM UMR-S 1138, “Cancer, Immune Control and Escape” Laboratory, Paris, France; ^2^Sorbonne Universities, Université Pierre et Marie Curie, UMR-S 1138, Paris, France; ^3^Université Paris Descartes, UMR-S 1138, Paris, France; ^4^Kennedy Institute of Rheumatology, University of Oxford, Oxford, United Kingdom

**Keywords:** adaptive immunity, antibody-induced immunogenic cell death, CD20, hematologic malignancies, immunotherapy, therapeutic monoclonal antibodies, vaccinal effect

## Abstract

Clinical responses to anti-tumor monoclonal antibody (mAb) treatment have been regarded for many years only as a consequence of the ability of mAbs to destroy tumor cells by innate immune effector mechanisms. More recently, it has also been shown that anti-tumor antibodies can induce a long-lasting anti-tumor adaptive immunity, likely responsible for durable clinical responses, a phenomenon that has been termed the vaccinal effect of antibodies. However, some of these anti-tumor antibodies are directed against molecules expressed both by tumor cells and normal immune cells, in particular lymphocytes, and, hence, can also strongly affect the host adaptive immunity. In addition to a delayed recovery of target cells, lymphocyte depleting-mAb treatments can have dramatic consequences on the adaptive immune cell network, its rebound, and its functional capacities. Thus, in this review, we will not only discuss the mAb-induced vaccinal effect that has emerged from experimental preclinical studies and clinical trials but also the multifaceted impact of lymphocytes-depleting therapeutic antibodies on the host adaptive immunity. We will also discuss some of the molecular and cellular mechanisms of action whereby therapeutic mAbs induce a long-term protective anti-tumor effect and the relationship between the mAb-induced vaccinal effect and the immune response against self-antigens.

## Introduction

Immunotherapy has now gained its place among the therapeutic arsenal of cancer therapies, based on the demonstration that tumors are under the surveillance of the host immune system (1), brought by studies performed on large cohorts of cancer patients with a high incidence case (2–4). In particular, monoclonal antibody (mAb) biotherapies have shown remarkable results in a significant number of cancer patients. A first category of antibodies are directed against tumor cells. It includes antibodies directed against tumor cells belonging to the hematopoietic lineage, lymphocytes (CD20, CD52, CD38, etc.), and myeloid cells (CD30, CD33, etc.). A number of other anti-tumor antibodies are directed against molecules expressed by a large variety of cell types (HER2/neu, EGFR, etc.). These antibodies are classically thought to work through a variety of mechanisms. It includes the inhibition of ligand binding to specific receptors, the blockade of receptor activation, thus interfering with signaling pathways, and/or through their ability to deplete tumor cells. Also, some antibodies block the neovasculature formation that accompanies tumor development [anti-vascular endothelium growth factor (VEGF), anti-EGFR, or anti-VEGFR2]. Moreover, another category of antibodies directed against immune checkpoint (ICP) molecules has emerged over the last decade, able to modulate the cellular and molecular microenvironment of tumors, as exemplified by the successful use of anti-CTLA-4 or anti-PD-1 antibodies.

The capacity of anti-tumor antibodies to deplete cancer cells has been largely documented *in vitro* and in preclinical animal settings. Antibodies exhibiting a human IgG1 Fc region (which represents a large proportion of antibodies used for cancer treatment) trigger Fc-dependent effector mechanisms [complement-dependent cytotoxicity (CDC), antibody-dependent cell cytotoxicity (ADCC), and phagocytosis]. The activation of the classical pathway of complement through the binding of C1q to the Fc portion of mAbs and the recruitment of Fcγ receptors (FcγRs) expressed by NK cells, neutrophils, monocytes, and macrophages lead to the formation and/or the release of effector molecules (membrane attack complex made of C5b-C9, perforin and granzymes, TNF-α, Reactive Oxygen Intermediates, etc.) that induce cell death. This has stimulated a lot of engineering efforts over the last 20 years, aimed at boosting effector mechanisms relying on the Fc region of IgG (5, 6).

Strikingly, reports based on clinical data and on *in vivo* animal models have suggested that antibody treatments leading to cell lysis and depletion could also induce a long-term anti-tumor response through the triggering of an adaptive memory response, a phenomenon that has been termed the “vaccinal” effect of antibody treatment (7–21). Anti-CA125- (8), anti-MUC1- (9), anti-HER2/neu- (10, 11), and anti-EGFR (12)-specific B and T cell responses have been reported in cancer patients following mAb therapy. Studies in murine models reported also that the therapeutic effect of anti-CD20 (13–16), anti-HER2/neu (17–20), or anti-EGFR (21) mAbs depends on the induction of an adaptive immune response and on the presence of T cells. The anti-HER2/neu studies revealed an antibody-mediated mechanism in which danger signals activate both innate and T cell-mediated immune responses (17–20). In addition, these studies showed that an immunological memory is required for tumor control and to enable animals to resist a tumor rechallenge (13–21). The idea that antibody treatment can lead to a long-lasting adaptive immune response in patients has therefore opened an exciting avenue for the manipulation of the host immune surveillance. Interestingly, chemotherapy that is often used in combination with therapeutic anti-tumor antibodies can also, in some circumstances, induce an immune adaptive response. A number of studies have launched the concept of immunogenic cell death (ICD) induced by chemotherapeutic drugs (22, 23) and have suggested that these drugs can induce an adaptive immune response against tumor cells. The molecular mechanisms of ICD induction involves the exposition of calreticulin (CRT) on the surface of the dying tumor cells, the release of danger signals such as the high-mobility group box 1 protein (HMGB-1) and ATP, leading to the processing of tumor antigens by stimulated dendritic cells (DCs) and to Tc1 polarization of CD8^+^ T lymphocytes (24).

However, a number of anti-tumor antibodies target molecules expressed by tumor cells belonging to the hematopoietic lineage and, hence, also target their normal cell counterparts, notably lymphocytes (anti-CD20, -CD52, -CD38, SLAMF7, etc.) and myeloid cells (anti-CD30, -CD33, etc.). These antibodies are mostly depleting antibodies and one can think, therefore, that it may impact the effects of mAb therapy on the long-term immune response of the patients. In patients with inflammatory/autoimmune diseases and in cancer patients, the iterative infusion of anti-lymphocyte depleting mAbs leads to a profound, selective, and, sometimes, long-lasting depletion of B and/or T cells. Quantitative and qualitative changes in B and T cell subsets and repertoires have been reported following reconstitution (25–33). Some patients with rheumatoid arthritis (RA) remain lymphopenic 12 years after alemtuzumab (anti-CD52) treatment, and the analysis of their peripheral T cell compartments shows that naïve and central memory T cell (T_CM_) numbers are reduced, while that of effector memory T cells (T_EM_) is similar to that of RA patients not treated with alemtuzumab (32). There is also an extensive literature concerning abnormalities of B cell repopulation after rituximab (anti-CD20) treatment, including a delayed recovery of circulating CD27^+^ memory B cells and/or changes in immunoglobulin repertoires, in patients with autoimmune diseases (RA, systemic lupus erythematosus, and active primary Sjögren’s syndrome) or with B cell non-Hodgkin lymphoma (B cell NHL) (25–31, 33). Moreover, B cell depletion after rituximab treatment in autoimmune disorders affects T cell differentiation and activation and provokes an increase in regulatory T cell (Treg) numbers (34–38).

Together, these different studies indicate that the consequence of antibody treatment on the host adaptive immunity is multifaceted. Some therapeutic mAbs, particularly mAbs directed against immune cells, could not only have overall long-term unwanted effects on T cell compartments but also favor the emergence of specific anti-tumor adaptive immune responses.

## Antibody Treatments Can Induce Specific Memory Cellular Responses

Studies have demonstrated that, although the clearance of CD20^+^ lymphoma cells or of HER2^+^ or EGFR^+^ carcinoma cells by anti-CD20 (13), anti-HER2/neu (17), or anti-EGFR (21) antibodies, respectively, requires innate immunity, a phase of tumor growth control involving antigen-specific T cell response is then established (13–21).

With regard to anti-CD20 therapy, this phenomenon was initially shown in our laboratory using an experimental setting where mouse lymphoma T cells expressing human CD20 (huCD20) molecules (EL4-huCD20) injected intravenously (i.v.) into C57Bl/6 mice were targeted with an anti-huCD20 antibody (13–15). In this preclinical model, the depletion of CD4^+^ T cells, both at the initiation of the treatment and upon tumor rechallenge in surviving mice, dramatically reduced the protective effect of the antibody (13). Interestingly, a lack of CD8^+^ T cells at the beginning of the treatment did not impair its efficacy. By contrast, these latter cells were required when animals were rechallenged with tumor cells (13). This long-term protective effect based on T cell immunity, specifically directed against CD20^+^ and not CD20^−^ tumor cells, could be reinforced by IL-2 treatment at the time of rechallenge (13). Importantly, the anti-CD20 treatment modified the phenotype of CD4^+^ T cells by preventing the expansion of pro-tumor CD4^+^ Treg cells and by inducing a polarization toward a Th1 phenotype through the IFN γ/IL-12 axis. This was associated with an expansion of a pool of memory CD4^+^ T cells in long-term surviving mice (14) as well as by a change in the CD4^+^/CD8^+^ T cell ratio. The vaccinal effect of this anti-CD20 therapy was also shown to be dependent on the presence of the Fc region of the antibody (13), and on the activation of myeloid DC producing IL-12 (14). It was further demonstrated by others, using transgenic mice that this vaccinal effect relies on the binding of anti-CD20 antibody to human FcγRIIa and FcγRIIIa (15). Thus, the use of a depleting mAb targeting an antigen such as CD20 enables the triggering of a cascade of events that leads to the setting of a long-term adaptive immunity against this molecule. A recent study explored the relative role of CD4^+^ and CD8^+^ T cells in the host adaptive immune response triggered by an anti-CD20 mAb. A model of immune-competent mice bearing syngeneic mouse B cell lymphoma (A20) and treated with a mouse anti-mouse CD20 mAb was set up (16). The authors observed in this model that CD8^+^ T cells, instead of CD4^+^ T cells as described in the EL4-huCD20 model, played an essential role in CD20 mAb-mediated tumor regression. Anti-CD20 mAb induced type I IFN production by macrophages that promoted DC-mediated cross-presentation and priming of tumor-specific CTL (16). Interestingly, anti-CD20 mAb treatment resistance and tumor relapse in this model were associated with a much higher percentage of CTLA-4 expressing Treg. Finally, CTLA-4 blockade could synergize with anti-CD20 treatment to overcome the resistance to the development of an adaptive immune response in this preclinical model (16).

The differences in the results about T cell compartments involved in the mAb-induced long-term protective effect observed between the latter study and the previous one might be related to the differences in the nature of tumor cells injected (syngeneic *vs* xenogeneic) and the injection route (subcutaneously *vs* i.v.). Although highlighting the potential vaccinal effect of therapeutic mAbs, mice models based on the inoculation with EL4-huCD20 tumor cells (13–15) present some limitations: (i) the huCD20 molecule is a xenoprotein which likely displays a weak immunogenicity, reinforcing the mAb-induced anti-tumor specific T cell responses in mice; (ii) the anti-huCD20 mAb used in the studies on the vaccinal effect (13–15) does not exhibit any cross-reactivity with mouse CD20 and, hence, does not deplete non-malignant mouse B cells, contrary to mouse syngeneic models where mouse anti-mouse CD20 are used or to rituximab treatment in FL patients. Of note, in the different studies described above, anti-CD20 mAbs are used as a single agent. This does not reflect anti-CD20 treatment usually indicated for initiation therapy in NHL patients, performed in combination with chemotherapy (cyclophosphamide, doxorubicin, vincristine, prednisone) (CHOP). It suggests that further studies in patients with cancer or autoimmune/inflammatory diseases and treated with rituximab only or in combination with other drugs could be of major interest to decipher the long-term impact of anti-CD20 mAbs on B and T cell compartments and on the emergence of specific anti-CD20 T cell responses. Some data suggest that anti-CD20 therapy is capable of reinforcing the priming of tumor-specific T cells in patients. First, *in vitro* experiments have shown that bone marrow-derived macrophages present an increased cross-priming function when cocultured with human B cell lymphoma cells in the presence of rituximab (16). Second, anti-idiotype T cell responses have been detected in some FL patients following rituximab therapy (39).

Several preclinical studies have shown the important role of the host adaptive immune system in the anti-HER2/neu mAb-mediated anti-tumor immunity using HER2/neu^+^ mouse tumor models (17–20). Park and his collaborators demonstrated that the anti-tumor activity of an anti-HER2/neu antibody is abrogated into Rag-1^−/−^ mammary tumor-bearing mice (17). Of note, the same anti-HER2/neu antibody treatment in immune-competent mice induced tumor-specific CD8^+^ T cells producing IFN-γ and a protective memory T cell response that could be evidenced when mAb-treated surviving animals were rechallenged with tumor cells (17). It has also been demonstrated in another preclinical investigation that CD8^+^ T cell depletion in HER2/neu^+^ tumor-bearing mice treated with a combination of antibodies directed against HER2/neu and death receptor 5 abrogates the anti-tumor protective effect (19). A recent study using mouse models of HER2/neu^+^ breast cancer and examining tumor samples from HER2/neu^+^ breast cancer patients revealed that IL-21 expression in tumor-infiltrating CD4^+^ T cells is enhanced following anti-HER2/neu mAb therapy and that IL21R expression on CD8^+^ T cells is required for optimal mAb efficacy (40). Mortenson et al. have also evaluated the role of CD4^+^ T cells using mice engrafted with tumor cells overexpressing HER2/neu and treated with an anti-HER2/neu antibody in conjunction with CD4 depletion or CD40L blockade. They have shown that, in addition to CD8^+^ T cells, CD4^+^ T cells are also essential for anti-HER2/neu antibody-mediated tumor regression. The role of CD4^+^ in this model was not limited to CD8^+^ T cells help, but was also dependent on the production of IFNγ by innate cells, leading to MHC II expression on the tumor cells used in the model, allowing direct recognition of these cells by CD4^+^ T cells (18). This result suggested that mAb treatment can favor a direct recognition by specific CD4^+^ T cells of tumor-associated antigens (TAAs) in complex with MHC II molecules at the surface of tumor cells. However, this observation is strongly tempered by the fact that, unlike MHC I molecules that are ubiquitously expressed in human nucleated cells, the expression of MHC II molecules on solid tumors is far more restricted. MHC II expression has been detected on a variety of human cancer types (including ovarian, colorectal and lung cancer, melanoma, and breast carcinomas) (41), but is highly dependent on the immunological environment and can be modulated by cytokines (41). Furthermore, the capacity of MHC II^+^ tumor cells to prime naive CD4^+^ T cells is dependent on their ability to process tumor-derived antigens (41, 42). In patients, several clinical studies have demonstrated the induction of specific anti-TAA T and B cell responses following mAb treatment (8–12). An induction of CA125-specific B and T cell responses after injection of a murine anti-CA125 mAb (used as an immunoscintigraphic agent in patients with ovarian cancer) has been observed and correlated with improved survival (8). Moreover, an increase in MUC1-specific T cells frequency has been observed in cancer patients treated with anti-MUC1 mAb (9). The analysis of metastatic breast cancer patient immunity before and after trastuzumab (anti-HER2/neu) therapy showed that the percentage of patients exhibiting specific anti-HER2/neu B and T cell responses was increased, and the frequencies of memory specific cells were higher following mAb treatment (10). Patients with head and neck cancer (HNC) who have been treated with cetuximab (anti-EGFR) also presented a dramatic increase in tumor-specific CTL cells, their activation being dependent on NK-DCs cross-talk (12).

Finally, one can argue that the vaccinal effect of therapeutic antibodies is induced in many different immune contextures, including infectious diseases (Table 1). It was shown that the treatment of FrCas(E) retrovirus-infected mice with a neutralizing mAb resulted in a strong long-lasting immunity (43). The authors demonstrated that the mAb therapy inhibited an expansion of immunosuppressive Treg, reinforcing antiviral CD8^+^ T cell responses (44). Likewise, a study examining efficacy of mAbs against the gag envelop protein of HIV showed that monkeys receiving the anti-gag treatment exhibited higher gag-specific T cell responses (45).

**Table 1:** Effects of monoclonal antibody (mAb) treatment on host adaptive immunity.

mAb target	mAb	Preclinical model/disease	Effects on adaptive immunity	Reference
gp70 (Friend leukemia virus envelope)	A9D41	gp70 positive Friend Leukemia cells (tumoral mouse model)	Induces anti-tumor humoral immunity and depletion of CD4 cells reduces the efficacy of A9D41 mAb	(46)
MUC1	BrevaRax (AR20.5)	MUC1^+^ tumors in cancer patients	Induces specific anti-MUC1 B and T cell responses	(9)
CA125	B43.13	Ovarian cancer patients	Induces specific anti-CA125 B and T cell responses	(8)
CD20	Rituximab	Non-Hodgkin lymphoma patients	Induces anti-idiotype T cell responses	(39)
CD20	CAT.13	Human CD20^+^ thymoma (mouse model)	Induces long-term protective T cell immunity	(13–15)
CD20	Mouse anti-mouse CD20	Syngeneic B cell lymphoma (mouse model)	Induces long-term protective T cell immunity	(16)
HER2/neu	7.16.4/Trastuzumab	HER2/neu^+^ tumor (mouse model) and biopsies from breast cancer patients	Induces anti-tumor memory T cell responses	(17–20, 40)
HER2/neu	Trastuzumab	Breast cancer patients	Induces specific anti-HER2/neu B and T cell responses	(10, 11)
EGFR	Cetuximab	Human EGFR^+^ tumor cells (mouse model)	Induces anti-tumor T cell responses	(21)
EGFR	Cetuximab	Head and neck cancer patients	Induces anti-EGFR T cell responses	(12)
FrCas(E)	667	Retroviral infection (mouse model)	Prevents regulatory T cell expansion and induces long-term protective immunity	(43, 44)

## Vaccinal Effect: How Does it Work?

Until recently, mAb therapy was viewed as a passive therapy acting rapidly and directly against tumor cells and was not classified as “biotherapy.” CDC and ADCC/ADCP exerted by cells from the innate immunity through the engagement of FcγR are considered to play an important role in the *in vivo* efficacy of anti-tumor antibodies both in preclinical tumor models and in treated cancer patients (47). The significant correlations of FcγR polymorphisms with the clinical outcome in patients treated with rituximab (48, 49), trastuzumab (50, 51), and cetuximab (52, 53) argue in favor of a role of FcγR^+^ immune cells activation in the clinical responses to mAb-based treatment. However, as other studies revealed no such associations in patients with breast cancer (54) and in follicular lymphoma (55, 56), it raises the possibility of additional immune mechanisms to account for the clinical benefit of mAb-based immunotherapy. Notably, the duration and strength of clinical responses following mAb treatment may be linked to the ability of tumor antigen-specific mAb to elicit an adaptive cellular immunity.

**Figure 1:**
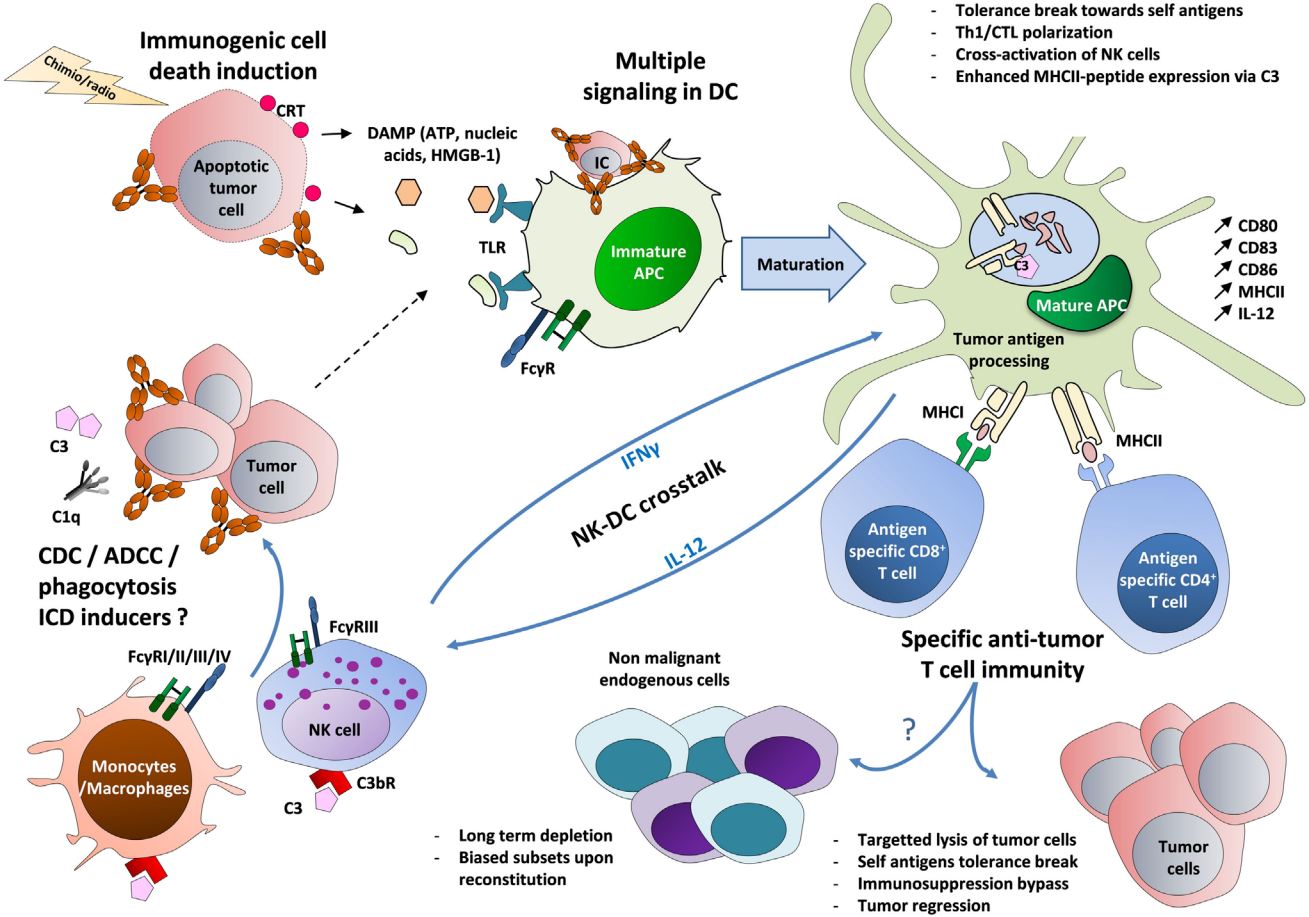
The circuits of the vaccinal effect of monoclonal antibodies in cancer. Tumor cells opsonized with antibodies recruit C1q molecule and FcγR-expressing innate cells, such as macrophages and NK cells (63–84). This leads to cell lysis and to the formation of cell debris through phagocytosis, ADCC, and CDC (5, 6). Immature DCs then capture the resulting immune complexes (made of Ag-containing tumor lysate and antibody) (57–61). In parallel, tumor cells treated by radiotherapy or chemotherapy may undergo ICD, leading to the exposure of CRT on the surface of dying cells and to the release of ATP and HMGB-1. The latter molecule triggers TLR-mediated inflammation (22–24). These multiple signals then lead to DC maturation (upregulation of MHC II, CD80, CD83, and CD86) and to the production of Th1-prone cytokines (IFNγ, IL-12) (85, 86). A tolerance break can occur, marked by the presentation of tumor-associated self-antigens on MHC I and MHC II, possibly reinforced by the capacity of C3 to enhance MHC II exposure (57–61, 87). The activation of IL-12-producing DCs could also be strengthened by a positive cross-talk with IFNγ-producing NK cells, leading to a stronger activation of both cell types (64, 65). Altogether, these mechanisms lead to the priming of self-reactive tumor-specific CD4^+^ and CD8^+^ T cells that can act back against tumor cells and eventually circumvent the pro-tumor immunosuppression (regulatory T cells, IL-10, TGF-β, etc.) (Table 1). These self-reactive T cells could also impact endogenous cells expressing the same targeted antigens, with a long-term depletion and biased subsets upon reconstitution (25–33). FcγRIV is only expressed in mouse on myeloid cells. ADCC, antibody-dependent cell cytotoxicity; APC, antigen-presenting cell; CDC, complement-dependent cytotoxicity; CRT, calreticulin; CTL, cytotoxic T lymphocyte; C3bR, receptor for the C3b complement fragment; DCs, dendritic cells; IC, immune complex; ICD, immunogenic cell death; DAMP, damage-associated molecular pattern; FcγR, receptors for the Fc region of IgG; HMGB-1, high-mobility group box 1 protein; MHC, major histocompatibility complex; TLR, toll-like receptor.

Various hypotheses may explain the induction of an adaptive immunity following antibody-mediated effector functions (Figure 1). A role for DCs present at the tumor site is supported by their ability to internalize immune complexes *via* activating FcγR and to promote efficient MHC II- and MHC I-restricted presentation of peptides from exogenous IgG-complexed antigens (57–59). It has been reported that anti-CD20 antibody-treated lymphoma cells are taken up and processed by DCs with subsequent cross-presentation of tumor-derived antigens to T cells (60). In a human glioma model, FcγR-dependent engulfment of cetuximab-coated glial tumor cells by DCs leads to an increased number of anti-tumor CD8^+^ T cells (61). Interestingly, DC cross-presentation of MHC I-restricted tumor peptides derived from exogenous immune complexes could be improved using blocking antibody directed against inhibitory FcγRIIb (58, 61). Moreover, the crucial role of FcγR-expressing DC has also been recently demonstrated when comparing a bispecific anti-G_D2_ x anti-G_D3_ antibody (BsAb) lacking the immunoglobulin Fc region *vs* the same trifunctional BsAb comprising an appropriate Fc region. Only the trifunctional antibody elicited a polyvalent, DC-dependent long lasting anti-tumor T cell response (62).

Several studies demonstrated that, upon mAb therapy, a cross-talk between NK cells and DC can occur (12, 63, 64). One of the most illustrative studies showed that cetuximab-activated NK cells could induce an IFNγ-dependent expression of DC maturation markers, of antigen processing machinery components such as TAP-1/2 and Th1-related cytokines, resulting in enhanced cross-presentation to CTL specific for EGFR-derived peptides (12). MAb-mediated NK-DC cross-talk may also favor antigen spreading by promoting DC maturation, tumor cells destruction, and CD8^+^ T cells priming. Moreover, in the same study, mature DCs stimulated mAb-activated NK cells in return, leading to an increased secretion of IFNγ (12). In a mouse model of C57Bl/6 mice injected i.v. with huCD20^+^ tumor cells and treated with an anti-huCD20 antibody, we have shown that IFNγ-producing NK cells, costimulatory molecules-expressing mature DC, and IL-12 production are critical for mAb-induced vaccinal effect (14). Recently, a study using the same model of anti-huCD20 mAb treatment of tumors in transgenic mice expressing human FcγR has demonstrated that the induction of anti-tumor adaptive immunity is dependent on the expression of FcγRIIA on DC and on FcyRIIIA-mediated ADCC (15). A recent study using a syngeneic model of B cell lymphoma in mice has shown that depletion of DC impairs the anti-tumor effect of anti-CD20 mAb (16). In this model, mAb treatment increased the cross-presentation of DC and the cross-priming of anti-tumor CD8^+^ T cells involved in tumor regression (16). Moreover, IL-12 and IFNγ production by NK cells enhanced the anti-tumor efficacy of trastuzumab in a model of murine colon adenocarcinoma (65).

The relationship between macrophages and tumors is complex as some of them exhibit anti-tumor activity (M1 macrophages) and others, termed M2 macrophages, secrete tumor growth factors and favor angiogenesis. In a clinical trial, the addition of cetuximab to bevacizumab (a humanized anti-VEGF-A mAb) plus chemotherapy resulted in a decreased progression-free survival in metastatic colorectal cancer. The authors could then show that M2 macrophages were the dominant FcγRIII^+^ population in the tumor microenvironment of these patients (66). However, macrophages are likely crucial to the efficacy of therapeutic antibodies thanks to their expression of different types of FcγR, enabling ADCP. There are several *in vitro* and *in vivo* evidence supporting macrophages as effectors of therapeutic antibodies in cancer. *In vitro* macrophage phagocytosis of tumor cells in response to anti-CD20 and anti-HER2/neu mAbs has been demonstrated in a number of studies using human macrophages (67–71). Interestingly, all human IgG subclasses, including isotypes which exhibit a low NK cell-mediated ADCC due to their poor binding to FcγRIIIa, have the potential to engage other FcγRs [FcγRI and FcγRIIa] expressed on macrophages and to stimulate macrophage-dependent phagocytosis (72). It has also been demonstrated that macrophage-mediated phagocytosis contributes to the therapeutic activity of anti-CD38 antibody in multiple myeloma and potentially other hematological tumors (73). *In vivo* studies using anti-CD20 mAbs have also demonstrated a crucial role of monocytes and macrophages as effectors of anti-tumor activity of antibodies, while depletion or defects in these populations induce impaired responses to mAb treatment (74, 75). Some studies have also shown that Kupffer cells in the liver immobilize and eliminate circulating tumor cells by engulfment of opsonized cells, thus probably contributing to the prevention of metastasis (75–77). Several clinical investigations have found that a high number of macrophages within tumors correlate with poor prognosis in many different types of cancers (78). However, the impact on prognosis of macrophage infiltration is dependent on the tumor microenvironment and on the combination of therapeutics as shown in different studies in lymphoma patients (79, 80). High tumor-associated macrophages number was associated with an adverse outcome in chemotherapy-treated patients, while it was associated with longer survival rates when chemotherapy was combined with rituximab (79). This study further implicates macrophages as important effectors for the therapeutic benefit of antibody treatment in patients and suggests that the balance between macrophages and mature DCs may provide some clues in the differential priming of the T cell response after antibody therapy. Interestingly, it has been reported that human macrophages and DCs for therapeutic use equally present TAA to CD8^+^ T cells after phagocytosis of γ-irradiated melanoma cells, indicating that macrophages can contribute to the recruitment of T cells as efficiently as DCs (81). Thus, one can hypothesize that phagocytosis of IC by FcγR^+^ macrophages can lead to an efficient CD8^+^ T cell cross-presentation. Of note, a role of type I IFN-producing macrophages in promoting cross-presentation of B cell lymphoma antigens by DCs has been recently reported (16).

Finally, the recruitment of complement molecules has also been reported after antibody infusions. The binding of C1q to the Fc region of mAbs can induce the lysis of target cells and promotes the recruitment of complement receptor-expressing effector cells (82, 83). These mechanisms are central in the anti-tumor activity of some therapeutic antibodies, such as ofatumumab, an anti-CD20 mAb that has been optimized for its capacity to bind C1q and to induce CDC (84). Interestingly, recent studies suggest that the C3 complement molecule has a role in the T cell response to apoptotic cell-associated antigens. The authors of the study showed that C3 acts as a chaperone protein in the intracellular processing of antigens derived from apoptotic cells and can, therefore, modulate T cell responses to self-antigens displayed on dying cells (87). This study underlines a new link between complement molecules recruited by immune complexes and the ability to promote presentation of tumor-derived antigens from dying cells to T cells.

## Antibody-Induced Expansion of Autoreactive T Cells? To be or Not to be Immunogenic…

Thus, all these data strongly support the hypothesis that the induction of a T cell response against mAb-targeted cells plays a key role in the long-term efficacy of mAb-based therapies, raising important questions about the specificity and the evolution of these responses all along the treatment. Furthermore, it is still unclear whether the induction of a long-term adaptive response that follows mAb treatment is related to the tumor nature of the target cells or whether it also applies to non-malignant cells depleted upon mAb treatment. Finally, since there are different forms of mAb-induced killing of target cells, it is unclear whether differences arise between normal and tumor cells in terms of immunogenicity once killed.

Most tumor antigens are self-antigens that elicit weak T cell responses if any as a consequence of immune tolerance. However, anti-tumor mAb treatment may at least transiently break tolerance to self-antigens expressed on tumor cells (17, 88). In cancer patients, treatment with trastuzumab and chemotherapy increases the frequency of CD4^+^ T specific for HER2/*neu* peptides already known to bind multiple HLA-DR molecules. Some of these peptides have recently been identified as epitopes exhibiting a high affinity to a variety of HLA class II molecules (10, 89, 90). Likewise, T cell responses to MUC1 were observed following anti-MUC1 mAb treatment using ELISPOT assays in response to stimulation with wild-type 31mer MUC1 peptide (9). Furthermore, the staining of CD3^+^ CD8^+^ T cells with a tetramer showed a higher frequency of EGFR_853–861_-specific T cells in HNC patients treated with cetuximab alone or in combination with chemotherapy than in cetuximab-naïve patients. It suggests that wild-type EGFR expressed on HNC cells could induce a specific immune response *in vivo* (12).

These results suggest that therapeutic mAbs treatment can lead to the expansion of preexisting T cells specific for non-mutated self-antigens. An emerging concept is that thymic deletion prunes but does not eliminate self-specific CD4^+^ and CD8^+^ T cells and that some self-peptide/MHC-restricted T cells can be detected in frequencies similar to those of the T cells specific for non-self antigens (91–96).

Interestingly, several studies of HLA ligandome or self-immunopeptidome have identified CD4^+^ and CD8^+^ T cell epitopes derived from B cell differentiation antigens (in particular, from CD19 and CD20 antigens) that could generate autoreactive cytotoxic T cell responses to B cell leukemia and lymphoma in patients with B cell malignancies (97–100). In one study, the authors analyzed the natural ligandome in primary chronic lymphocytic leukemia (CLL) patients by immunoaffinity purification of HLA-I and HLA-II molecules from PBMC followed by liquid chromatography/mass spectrometry analysis of HLA ligands. This analysis allowed the identification of non-mutated tumor-associated T cell self-antigens exclusively and frequently found in the HLA ligandome of CLL cells, by comparison with the HLA ligand proteomes of healthy donors. Furthermore, they evidenced an immune recognition by T cells of these self-antigens in CLL patients (99). Moreover, a recent report has demonstrated the role of T cells bearing low-avidity TCR for self-antigens in the immune surveillance of spontaneous lymphoma B cells that express MHC molecules presenting self-peptides in conjunction with high levels of costimulatory molecules and Fas (101). In line with these observations, T cells directed against tumor B cells could be expanded from tumor and peripheral blood of patients with B cell NHL (102). In hematological diseases, endogenous preexisting T cell response directed against tumor-specific clonal immunoglobulin expressed by lymphoma B cells [idiotype (Id)] has been used as a target for active immunotherapy (103). In addition, it has been shown that peptides derived from B-cell receptor pathway components are presented in the context of MHC I and MHC II molecules in patients with lymphoma (104). The CD20 molecule itself could give rise to self-peptides targeted by T cells. A strategy to detect and expand allo-MHC-restricted T cells reactive to self-tumor antigens resulted in the detection of 37 non-mutated epitopes from CD20 and myeloperoxydase (100).

Of note, a large majority of cancer patients are treated with tumor-targeting mAbs in combination with chemotherapy or radiotherapy. Thus, one can think that the capacity of mAb treatment to induce adaptive immune responses against self-antigens could be related to inflammatory signals provided by chemotherapy or radiotherapy-induced ICD. The molecular mechanisms of ICD implicate the exposure of CRT on the cell surface, the secretion of ATP, and the release of the non-histone chromatin-binding protein HMGB-1. The emission of these damage-associated molecular patterns (DAMPs) is associated with the secretion of immunostimulatory cytokines such as type I IFN. Such DAMPs are then able to recruit antigen-presenting cells, including DCs, to the site of ICD and promote dead cell-associated antigens presentation to CD4^+^ and CD8^+^ T cells resulting in the priming of a robust cellular response (85). Several drugs, including various chemotherapeutics commonly used in the clinic (doxorubicin, mitoxantrone, bleomycin, bortezomib, cyclophosphamide, oxaliplatin, etc.), have the ability to provoke ICD (86). However, there is growing evidence that some therapeutic mAbs, as single agents, could also be ICD inducers. In a mouse model of mammary tumors where animals were treated with an anti-HER2/neu antibody, the release of HMGB-1 was essential for antibody-mediated tumor regression. Based on the results obtained in Myd88^−/−^ mice, the authors suggested that anti-HER2/neu antibody could induce HMGB-1 release in the tumor microenvironment, which enhanced innate responses *via* the MyD88 pathway and promoted the priming of adaptive immune cells, leading to an increased tumor clearance (17). Moreover, 7A7, an anti-murine EGFR mAb, could elicit strong tumor-specific CTL responses in hosts by inducing ICD of tumor cells in an Fc-independent manner (105). Interestingly, a recent study has shown that programmed-cell death induced by the type II anti-CD20 antibody obinutuzumab (GA-101) is associated with the release of significant levels of DAMP that could enhance DC maturation and subsequent T cell activation (106). Moreover, a recent study reported that both obinutuzumab and rituximab induced HMGB-1 release from diffuse large B-cell lymphoma (DLBCL) cells after a 4-h treatment (107). The same study showed that treatment with rituximab plus cyclophosphamide, doxorubicin, vincristine, and prednisone, but not CHOP alone, significantly increased plasma HMGB-1 and decreased IL-10 concentrations in DLBCL patients. Furthermore, the conditioned medium from rituximab-treated DLBCL cells could induce DC maturation and increased their capacity to activate T cell responses (107). In line with these observations, long-term complete remissions have been reported after single-agent rituximab treatment in different clinical trials in patients with follicular lymphoma (108, 109).

## Concluding Remarks

Therapeutic mAbs used in oncology, inflammatory, and/or autoimmune disorders can exert depleting activity against target cells by inducing direct apoptosis and/or by recruiting effector cells from the innate immunity. Besides delayed recovery of target cells and impaired reconstitution of depleted populations in terms of frequencies and phenotype, lymphocytes depleting-mAb treatment could also have dramatic consequences in the network of cells from the adaptive immune compartment. As expected, the depletion of one particular immune population could have bystander effects on the homeostasis and the functions of other non-targeted immune compartments. This effect has been extensively described in autoimmune disorders treated with rituximab, where B cell depletion induces dramatic changes in T cell compartments.

Moreover, as depleting mAbs are potentially ICD inducers, it is likely that an adaptive immune response against target cells could be mounted following mAb treatment. Different preclinical studies (mostly in models with tumor-bearing mice treated with anti-tumor mAbs), strengthened by a few observations in human clinical studies, have shown that therapeutic antibodies have a vaccinal effect likely responsible for the long-lasting clinical responses that have been observed. These long-lasting anti-tumor effects are mediated by CD4^+^ and CD8^+^ T cells. The role of FcγRs in the induction of this adaptive immune response following mAb therapy supports further studies to carefully examine the impact of the structural properties of therapeutic mAbs on their ability to elicit strong adaptive immune responses. In particular, it is now well established that Fc sequences as well as the nature of Fc-CH2 domain-N-linked oligosaccharide have an impact on IgG/FcγR interactions and, thus, could influence the vaccine effect of mAbs. Interestingly, Fc domain variants of anti-HIV-1 broadly neutralizing antibodies that exhibited enhanced binding capacity for activating human FcγR, such as FcγRIIa and FcγRIIIa, also presented an augmented *in vivo* protective activity in a model of HIV-infected humanized mice compared to wild-type or FcγR null binding variants. In this study, Fc-optimized variants induced faster and sustained reduction in viral load, with a significantly higher proportion of infected mice demonstrating viremia suppression (110).

This novel paradigm of a vaccinal effect of therapeutic antitumor antibodies should lead us to reexamine how antibody efficacy could be reinforced by the use, possibly in combination, of molecules impacting the immune surveillance. Among them are antibodies antagonizing ICP molecules that play a key role in the inhibition of the anti-tumor immune responses (e.g., PD-1, CTLA-4, LAG-3, TIM3, etc.) or antibodies that target immunostimulatory molecules (e.g., OX40, GITR, CD137, etc.) (111, 112). Furthermore, it has been suggested that the use of radiolabeled antibodies in combination with maintenance rituximab therapy in lymphoma may lead to an increase in complete response, associated with an increased recruitment of T cell subsets (113).

Finally, there are currently a number of scattered data reported in the literature indicating that mAb treatment may result in inflammatory cell death that could lead to a targeted adaptive response. The induction of an ICD could be one of the mechanisms by which antibodies activate a long-term adaptive immunity against tumor cells and possibly normal cells. The latter is an important issue as it is still unclear whether a long-term adaptive immunity can be achieved when non-malignant cells are targeted with depleting mAbs, a situation found during the treatment of autoimmune pathologies. Various factors, such as the intrinsic immunogenicity of cells, their activation status, and the nature of the cell death pathway that is engaged, are all critical to determine whether cell death is immunogenic or not. Thus, monitoring adaptive immune responses after therapeutic mAb treatments might provide a further insight into the vaccine effect of mAb treatment, its impact on endogenous cell reconstitution, and its role in tumor control or relapse in high- or low-responder patients with cancer.

## Author Contributions

CD, SS, and J-LT conceived and wrote the manuscript. NJ and BM critically reviewed the manuscript. All the authors fully agreed with the manuscript content.

## Conflict of Interest Statement

The authors declare that the research was conducted in the absence of any commercial or financial relationships that could be construed as a potential conflict of interest.
